# Subclavian artery avulsion following blunt trauma: A case report and literature review

**DOI:** 10.1016/j.ijscr.2019.07.061

**Published:** 2019-07-25

**Authors:** Adel Elkbuli, Saamia Shaikh, Mark McKenney, Dessy Boneva

**Affiliations:** a Department of Surgery, Kendall Regional Medical Center, Miami, FL, United States; b University of South Florida, Tampa, FL, United States

**Keywords:** Subclavian artery, Avulsion, Subclavian artery bypass, Endovascular balloon occlusion, Vascular surgery, Trauma outcomes

## Abstract

**Introduction:**

Subclavian artery injuries are rare and are associated with a high morbidity and mortality. The majority of patients with blunt trauma to the subclavian artery succumb to their injury before reaching a hospital. In-hospital mortality remains high.

**Presentation of case:**

We report the case of a 30-year-old male who presented with complete traumatic subclavian artery avulsion after a motorcycle collision. He presented in hemorrhagic shock. Temporary hemostatic control was achieved with endovascular balloon occlusion followed by operative intervention.

**Discussion:**

Prompt diagnosis and meticulous management including early transfusion, when indicated, are necessary to salvage both the patient’s life and limb from such severe injuries. Intra-operative diagnosis of subclavian artery injury is most common due to the hemodynamic instability of most patients with such injuries; however, conventional angiography and computed tomography angiography are useful diagnostic modalities as well. Temporizing measures such as endovascular balloon occlusion to obtain vascular control may serve as a valuable adjunct to surgical repair.

**Conclusion:**

Repair of a subclavian artery injury is challenging for even the most experienced of surgeons. Endovascular techniques can be therapeutic or used as an adjunct to control bleeding ad allow an open repair.

## Highlights

• Subclavian artery injuries are rare, highly fatal, and constitute less than 2% of all vascular injuries.
• Surgical repair of these injuries can be technically challenging due to their anatomic location.
• Therapeutic approaches include surgical repair, endovascular repair, or a hybrid approach depending on the severity of the injury and the patient’s hemodynamic status.

## 1 Introduction

There is a fortress of protection around the subclavian vessels which makes injury relatively uncommon. However, it is this very fortress that makes surgical management challenging due to difficulty with accessibility and exposure [1]. Additionally, the proximity of the subclavian artery to a number of delicate structures makes the occurrence of concurrent injuries, which significantly increase morbidity and mortality, common [2]. Traumatic injury to the subclavian artery is usually the result of a penetrating injury with blunt injury occurring infrequently [3,4]. When blunt force results in injury to the subclavian artery it usually results in middle or distal subclavian artery injuries. Proximal subclavian artery injury following blunt force trauma is exceptionally rare due to its anatomic location inside the chest [3,5]. Diagnosis of subclavian artery injuries on physical examination can be difficult because the existence of collateral flow can prevent any obvious symptoms of ischemia and because of the resultant rapidly deteriorating status for which aggressive resuscitation takes precedence [4]. We present a case of a patient with blunt traumatic proximal subclavian artery avulsion that was successfully managed with a hybrid endovascular and open technique.

## 2 Case presentation

**Fig. 1:**
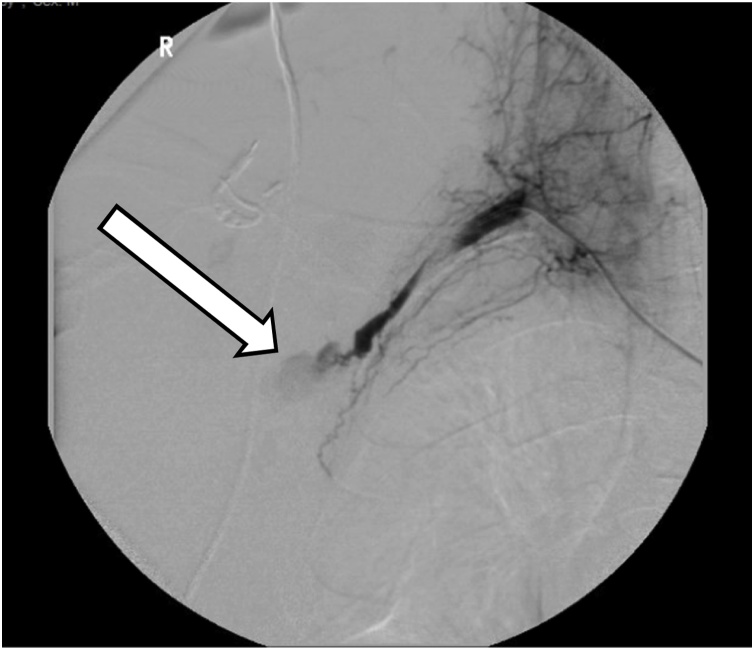
Right subclavian artery avulsion and extravasation localizing the precise location of complete transection of the right subclavian artery.

**Fig. 2:**
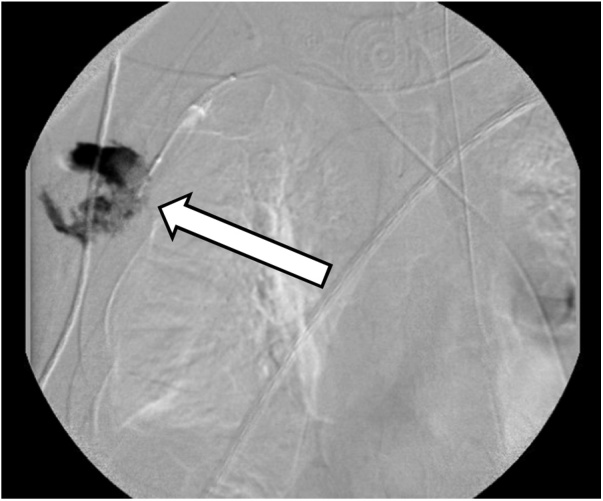
Local contrast extravasation as a result of complete transection of the right subclavian artery.

**Fig. 3:**
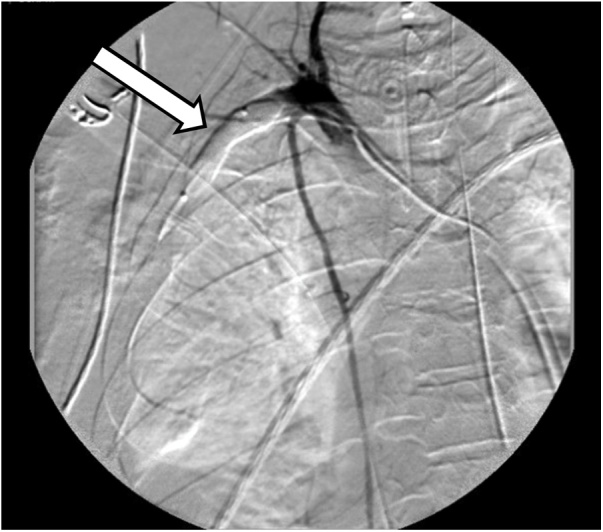
Endovascular balloon at the right proximal subclavian artery.

**Fig. 4:**
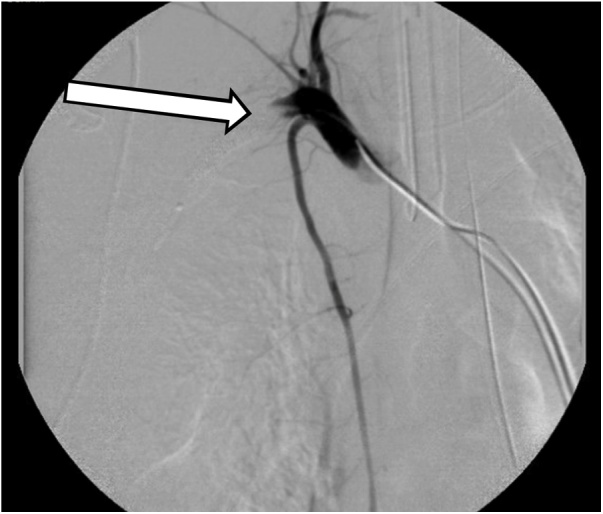
Balloon deployed at the proximal end of the right subclavian artery. Also depicted above is the right common carotid artery and the right internal mammary artery intact just proximal to the balloon.

**Fig. 5:**
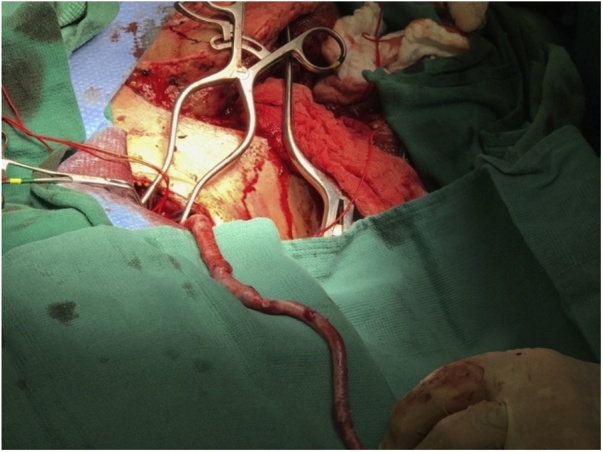
Right common carotid artery to right axillary artery bypass using cadaver vein graft.

A 30-year-old helmeted male presented to our trauma center following a high-speed motorcycle collision. He was ejected and landed twenty-five feet from his motorcycle. On arrival, he had a systolic blood pressure of 80 mmHg. A right-sided chest tube was placed for clinically diagnosed tension pneumothorax, with significant air but minimal blood returned. Focused assessment with sonography in trauma (FAST) exam was negative for intra-abdominal fluid/bleeding. With resuscitation efforts the patient’s hemodynamics improved. On secondary examination the patient’s right chest wall and shoulder were swollen. The radial pulse on the right was significantly reduced compared to the left. Due to high suspicion for an underlying vascular injury and his improvement in hemodynamics the patient was taken to the interventional radiology suite for imaging. Angiography revealed complete avulsion of the right subclavian artery (Figs. 1 & 2 ). An attempt at endovascular repair was made, however, the guide-wire could not be bridged across the avulsion. A balloon catheter was deployed at the proximal end of the subclavian artery, providing more secure temporary hemorrhage control (Figs. 3 & 4 ). The patient was taken to the operating suite for open exploration and repair of the right subclavian artery. A delto-pectoral approach was utilized. Dissection revealed that primary anastomosis was not possible due to the destruction of the proximal subclavian. Proximal and distal control was obtained with ligation and then the balloon occlusion was removed. Further dissection also revealed complete avulsion with thrombosis of the subclavian vein, which was also ligated. A right common carotid artery to axillary artery bypass was performed using a cryopreserved saphenous vein allograft (CryoLife, Inc. Kennesaw, GA) (Fig. 5). To mitigate the risk of thrombosis formation, intraoperative heparin was used for the duration of the surgery at 500 units per hour. After completion of the anastomosis, angiography of the right upper extremity revealed uninterrupted flow without defect, confirming patency of the vein graft and successful re-vascularization. The patient was taken to the intensive care unit (ICU) in critical condition.

He improved in the ICU and resolved his shock state. The following day, subcutaneous enoxaparin for venous thromboembolism prophylaxis was initiated and he was started on dual antiplatelet therapy with oral aspirin and clopidogrel for graft health and the heparin drip was discontinued. He continued to have a strong pulse in both upper extremities. He was discharged in stable condition and follow up at 2, 4 and 12 weeks revealed a patent bypass. This work has been reported in line with the SCARE criteria [6].

## 3 Discussion

Subclavian artery injuries associated with blunt trauma comprise less than 2% of all vascular injuries [2,5,7]. Of all patients with a blunt subclavian artery injury, only about 15% arrive to the hospital alive [5,7]. For the group arriving alive, in-hospital mortality rates are approximately 30% [8]. Morbidity and mortality in these patients is also dependent on additional injuries, which are common. In a multicenter, retrospective review by the Western Trauma Association, 13% of patients also had a subclavian vein injury, 4% of had an associated vertebral artery injury, and 1% of patients had an associated carotid artery injury [8]. Cox et al. reported that complications related to subclavian artery injuries in their series was dependent on hemodynamic status at the time of presentation. Death occurred in 46% of patients with blunt subclavian artery injury who arrived in shock [2].

Blunt injury to the subclavian artery is usually the result of a deceleration injury which transmits a tremendous amount of force to the upper extremities, neck, and chest [9,10]. Examples of such instances include motor vehicle crashes, motorcycle collisions, and falls from height [9]. In the hemodynamically stable patient, diagnosis can be made using angiography, or increasingly, with computed tomography angiography [11]. The diagnosis is also often made intra-operatively in many cases as patient hemodynamic instability does not allow for imaging. It is also not uncommon for these injuries to be initially overlooked as extensive collateral flow prevents any signs of ischemia. As such, the presence of distal pulses does not preclude subclavian artery injury [2].

Management approaches include open surgical repair, endovascular repair, or a hybrid approach. While open surgical repair has been the standard approach to date, endovascular and hybrid approaches are becoming more common [8]. The reason for this is twofold. First, obtaining adequate distal and proximal exposure of the subclavian artery is an intricate process and can be quite demanding when time is of the essence. Deep dissection is usually required to achieve sufficient exposure and surgeons must be extremely vigilant and not disturb or damage surrounding structures during the procedure. Second, endovascular repair carries with it numerous technical advantages including remote access to precisely the desired location [12]. Even if the injury is not amenable to endovascular repair, a balloon catheter can be deployed as a temporizing measure until definite surgical repair can be performed [13].

In a 13-year retrospective review, it was reported that when the subclavian vein had been injured along with the subclavian artery, surgeons usually sacrificed the vein, ligating it. Additionally, 13.1% of subclavian and axillary arteries were ligated because of the severity of the concomitant injuries, for which treatment took precedence [2,9]. While doing so may be life-saving it is not without consequence and can significantly increase morbidity. Using a hybrid approach can make surgery safer for patients as it has the potential to give surgeons the upper hand against time during an open repair, but this is only the case in hemodynamically stable patients. Hemodynamic instability usually warrants immediate operative intervention.

Common surgical approaches include supraclavicular, infraclavicular, supraclavicular/infraclavicular, trap-door, thoracotomy, median sternotomy, and limited sternotomy [3,14]. Surgical repair techniques reported in the literature include primary repair with end-to-end anastomosis, lateral arteriorrhapy, ligation, autologous or prosthetic vein graft, and vein patch angioplasty [3,10,14,15]. In the present case, the decision was made to use a cryopreserved saphenous vein allograft. An allograft was particularly appealing for our trauma patient with concomitant injuries, as it obviated the need for an additional harvesting procedure, preventing scarring at the donor site as well as minimizing operative time, and also because it allowed for immediate reconstruction. In addition to its “off the shelf” availability, the cadaveric vascular allograft also appears and handles similarly to autogenous vein grafts [16].

Furthermore, trauma patients often present with tenuous hemodynamics and the risks of initiating prophylactic anticoagulation must be delicately balanced with the potential harms of hemorrhagic complications. In doing so, a number of factors are taken into account including the acuteness of the trauma and the nature of the patient’s injuries. While our patient presented with injuries which could have potentially increased his bleeding risk, he was stabilized prior to initiation of the heparin drip and thus was deemed to be low-risk for bleeding complications.

Additionally, a management option in patients with other co-existing life-threatening injuries is to divert all attention from the limb to saving the patient’s life [17]. While such an approach obviously holds merit, it can carry with it long-lasting effects for the patient, such as limb loss. For our patient angiography prior to surgical intervention pinpointed the exact location of the injury, which was very proximal, and prevented blind dissection in the operating room. The endovascular balloon not only achieved temporary hemostatic control but also the inflated balloon served as a guide during dissection of the proximal artery injury.

## 4 Conclusion

Subclavian artery injury is rare and is associated with a high morbidity and mortality. Surgeons must have a high index of suspicion for subclavian artery injuries, especially following deceleration injuries, in order to timely detect such injuries. Angiography prior to operative intervention can identify the precise location of the injury. Endovascular techniques can be therapeutic or used as an adjunct to control bleeding and allow for a more controlled surgical approach.

## Sources of funding

None.

## Ethical approval

This is a case report study. Informed written consent has been obtained and all identifying information is omitted. This work has been conducted in compliance with institutional ethical standards.

## Consent

Informed written consent has been obtained and all identifying information is omitted.

## Author contribution

Adel Elkbuli, Saamia Shaikh, Dessy Boneva, Mark McKenney– Conception of study, acquisition of data, analysis and interpretation of data.

Adel Elkbuli, Dessy Boneva, Saamia Shaikh - Drafting the article.

Dessy Boneva, Mark McKenney – Management of case.

Adel Elkbuli, Saamia Shaikh, Dessy Boneva, Mark McKenney – Critical revision of article and final approval of the version to be submitted.

## Registration of research studies

This is a case report study.

## Guarantor

Dessy Boneva.

Mark McKenney.

## Provenance and peer review

Not commissioned, externally peer-reviewed

None.

## Declaration of Competing Interest

None.
